# A metabolic model of *Lipomyces starkeyi* for predicting lipogenesis potential from diverse low-cost substrates

**DOI:** 10.1186/s13068-021-01997-9

**Published:** 2021-07-01

**Authors:** Wei Zhou, Yanan Wang, Junlu Zhang, Man Zhao, Mou Tang, Wenting Zhou, Zhiwei Gong

**Affiliations:** 1 School of Chemistry and Chemical Engineering, Wuhan University of Science and Technology, 947 Heping Road, Wuhan, 430081 People’s Republic of China; 2 State Key Laboratory Breeding Base of Dao-Di Herbs, National Resource Center for Chinese Materia Medica, China Academy of Chinese Medical Sciences, Beijing, 100700 People’s Republic of China; 3 HuBei Province Key Laboratory of Coal Conversion and New Carbon Materials, Wuhan University of Science and Technology, Wuhan, 430081 People’s Republic of China

**Keywords:** *Lipomyces starkeyi*, Metabolic model, Flux balance analysis, Triacylglycerol, Theoretical lipid yield

## Abstract

**Background:**

*Lipomyces starkeyi* has been widely regarded as a promising oleaginous yeast with broad industrial application prospects because of its wide substrate spectrum, good adaption to fermentation inhibitors, excellent fatty acid composition for high-quality biodiesel, and negligible lipid remobilization. However, the currently low experimental lipid yield of *L. starkeyi* prohibits its commercial success. Metabolic model is extremely valuable to comprehend the complex biochemical processes and provide great guidance for strain modification to facilitate the lipid biosynthesis.

**Results:**

A small-scale metabolic model of *L. starkeyi* NRRL Y-11557 was constructed based on the genome annotation information. The theoretical lipid yields of glucose, cellobiose, xylose, glycerol, and acetic acid were calculated according to the flux balance analysis (FBA). The optimal flux distribution of the lipid synthesis showed that pentose phosphate pathway (PPP) independently met the necessity of NADPH for lipid synthesis, resulting in the relatively low lipid yields. Several targets (NADP-dependent oxidoreductases) beneficial for oleaginicity of *L. starkeyi* with significantly higher theoretical lipid yields were compared and elucidated. The combined utilization of acetic acid and other carbon sources and a hypothetical reverse β-oxidation (RBO) pathway showed outstanding potential for improving the theoretical lipid yield.

**Conclusions:**

The lipid biosynthesis potential of *L. starkeyi* can be significantly improved through appropriate modification of metabolic network, as well as combined utilization of carbon sources according to the metabolic model. The prediction and analysis provide valuable guidance to improve lipid production from various low-cost substrates.

**Supplementary Information:**

The online version contains supplementary material available at 10.1186/s13068-021-01997-9.

## Background

Microbial lipid has garnered much attention recently as it can be served as promising feedstock for edible oils, functional polyunsaturated fatty acids, oleochemicals, and biodiesel [1]. Oleaginous species routinely accumulate large amount of intracellular lipid under nutritional restriction, especially nitrogen starvation [2]. *Lipomyces starkeyi* is an excellent lipid producer featuring wide substrate spectrum, good tolerance to fermentation inhibitors, excellent fatty acid composition of lipid for high-quality biodiesel, and negligible lipid remobilization. A variety of low-cost materials including lignocellulosic biomass, starch materials, biodiesel derived glycerol, volatile fatty acids, molasses, and sewage sludge have been applied for lipid production by *L. starkeyi* [1, 3]. Especially, lignocellulosic hydrolysates have been directly utilized for lipid production without detoxification by *L. starkeyi*, which is of great significance for the commercial success [4, 5]. *L. starkeyi* exhibits high robustness to the major lignocellulosic inhibitors including acetic acid, furfural, and 5-hydroxymethylfurfural (HMF) and these agents even could be metabolized by the yeast [5]. In addition, *L. starkeyi* scarcely consumes the cellular lipid although the nutrients are completely exhausted compared with other oleaginous species, which is beneficial for the preservation [6].

High effective genetic transformation system is crucial for improving the oleaginicity of oleaginous yeasts. Recently, a variety of genetic transformation methods including lithium acetate-mediated transformation, PEG-mediated spheroplast transformation, agrobacterium-mediated transformation, and electroporation transformation have been established for *L. starkeyi* [7–10]. A site-directed gene knockout strategy has been reported in *L. starkeyi* NRRL Y-11558 [11]. The development of synthetic biology approaches, coupled with the omics technologies [12–14], has continuously deepened the understanding of lipid metabolism of *L. starkeyi* [3].

Metabolic model has been widely used in many fields including industrial biotechnology [15, 16]. The genome-scale metabolic model is convenient to predict biological capabilities and provide guidance for strain improvement. In recent years, a series of software have been developed to facilitate the automated and semi-automated construction of metabolic model [17]. Interestingly, genome-scale metabolic models of *Yarrowia lipolytica*, *Rhodotorula toruloides*, and *Cutaneotrichosporon oleaginosus* have been established to systematically analyze the lipid metabolism [18–20]. Small-scale metabolic model has been constructed in favor of some special purposes as the construction of the genome-scale metabolic model is very time-consuming and laborious. For example, Bommareddy and co-workers constructed a small-scale metabolic model of *Rhodosporidium toruloides* to evaluate the lipid production potential of several carbon sources [21]. A revised small-scale model containing 93 metabolites, 104 reactions, and 3 cell compartments was reconstructed by Castañeda and co-workers for more accurate prediction [22]. Tang and co-workers constructed a small-scale metabolic model of *C. oleaginosum* to evaluate the lipogenesis potential of chitin-derived carbon sources [23].

**Fig. 1:**
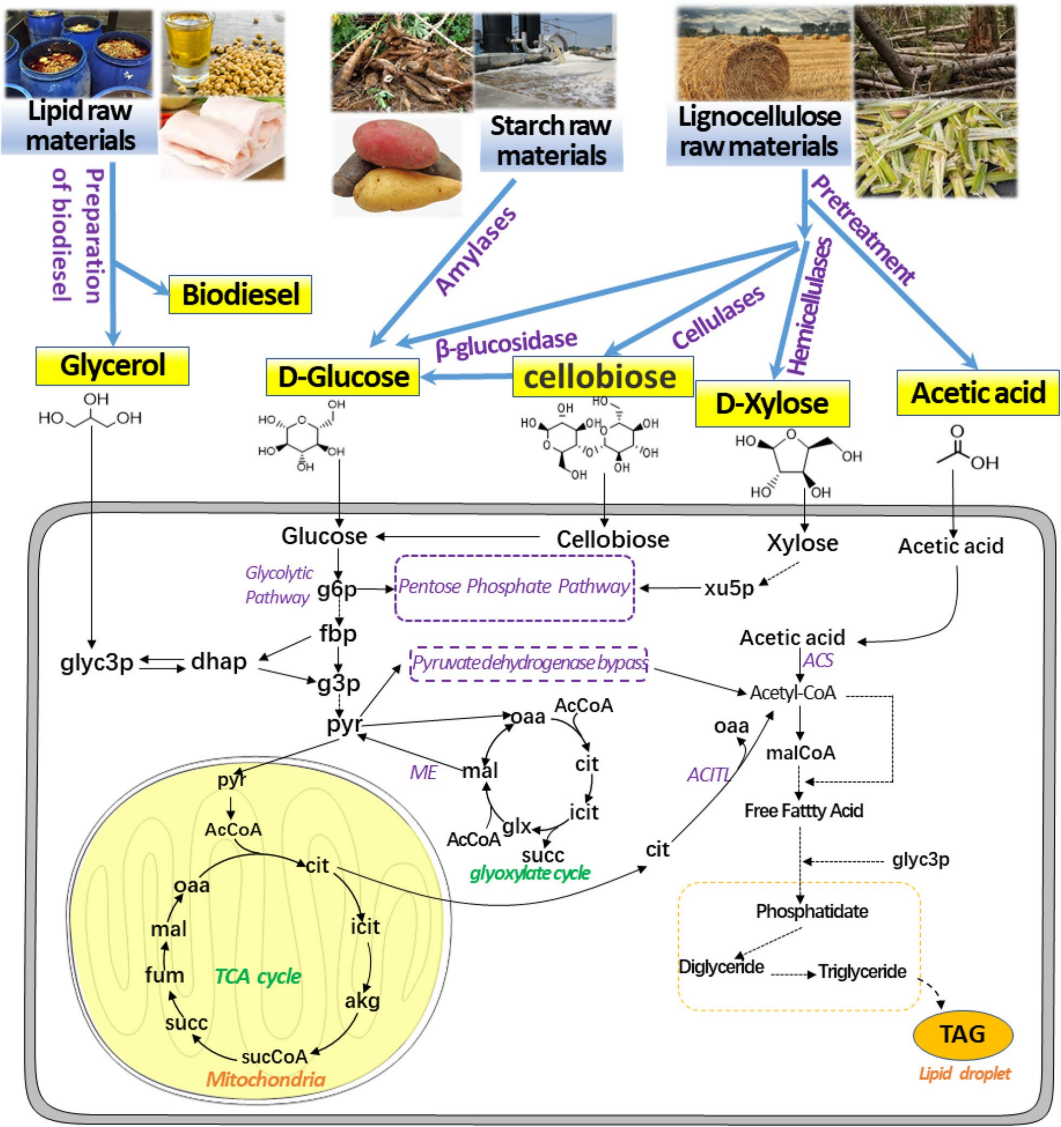
The metabolic pathways of lipid biosynthesis by *L. starkeyi* from a variety of carbon sources originated from diverse low-cost substrates

Glucose, xylose, cellobiose, glycerol, and acetic acid originated from a variety of low-cost substrates can be metabolized for lipogenesis by *L. starkeyi* (Fig. 1). However, the experimental lipid yields were merely ranging from 0.08 to 0.18 g/g as summarized in Table 1 [4, 24–32]. In this study, a small-scale metabolic model of *L. starkeyi* NRRL Y-11557 was constructed based on the genome annotation information. Flux balance analysis (FBA) was performed to calculate the theoretical lipid yields of a variety of carbon sources originated from low-cost substrates. Several targets (NADP-dependent oxidoreductases) were evaluated for improving the potential of lipid biosynthesis in *L. starkeyi*. The strategy of combined utilization of carbon sources mixture was investigated for improving the theoretical lipid yield. In addition, a hypothetical reverse β-oxidation (RBO) pathway was added to the model to estimate the lipid synthesis potential. This study provides valuable guidance and promising strategy to significantly improve lipid accumulation capacity from low-cost substrates.

**Table 1:** Lipid production from a variety of carbon sources by *L. starkeyi*

Strain number	Substrates	Culture	Biomass	Lipid content	Lipid yield	References
		Mode	(g/L)	(%, w/w)	(g/g)	
CBS 1807	Glucose	FB	13.30	60.47	0.11	[4]
DSM 70,295	Glucose	SF	9.4	68	ND	[24]
	Sewage sludge	SF	9.27	35.6	ND	
NRRL Y-11557	Glucose	SF	8.7	30	0.1	[25]
	Potato starch	B	11.7	40.3	0.16	
CBS 1807	Glucose, fructose, sucrose	SF	12.28	47.3	0.13	[26]
	Sweet sorghum juice	SF	21.69	29.5	0.08	
NRRL Y-11557	Raw glycerol	SF	5.74	50.49	0.13	[27]
NRRL Y-11557	Glucose	SF	18.28	54.85	0.17	[28]
	Corn stover hydrolysate	SF	24.63	38.07	0.14	
ATCC 58,680	Glucose and xylose	SF	9.43	56	0.18	[29]
CBS 1807	Glucose	SF	18.71	28.9	ND	[30]
	Xylose	SF	12.28	77.5	ND	
	Cellobiose	SF	11.73	35.6	ND	
NRRL Y-11557	Glucose	B	5	46	0.177	[31]
DSM 70,295	Crude glycerol	FB	32.7	55.9	0.15	[32]

SF: shake flask, B: bioreactor, FB: fed-batch

ND: no data reported

## Results and discussion

### Construction of the small-scale metabolic model of *L. starkeyi*

**Fig. 2:**
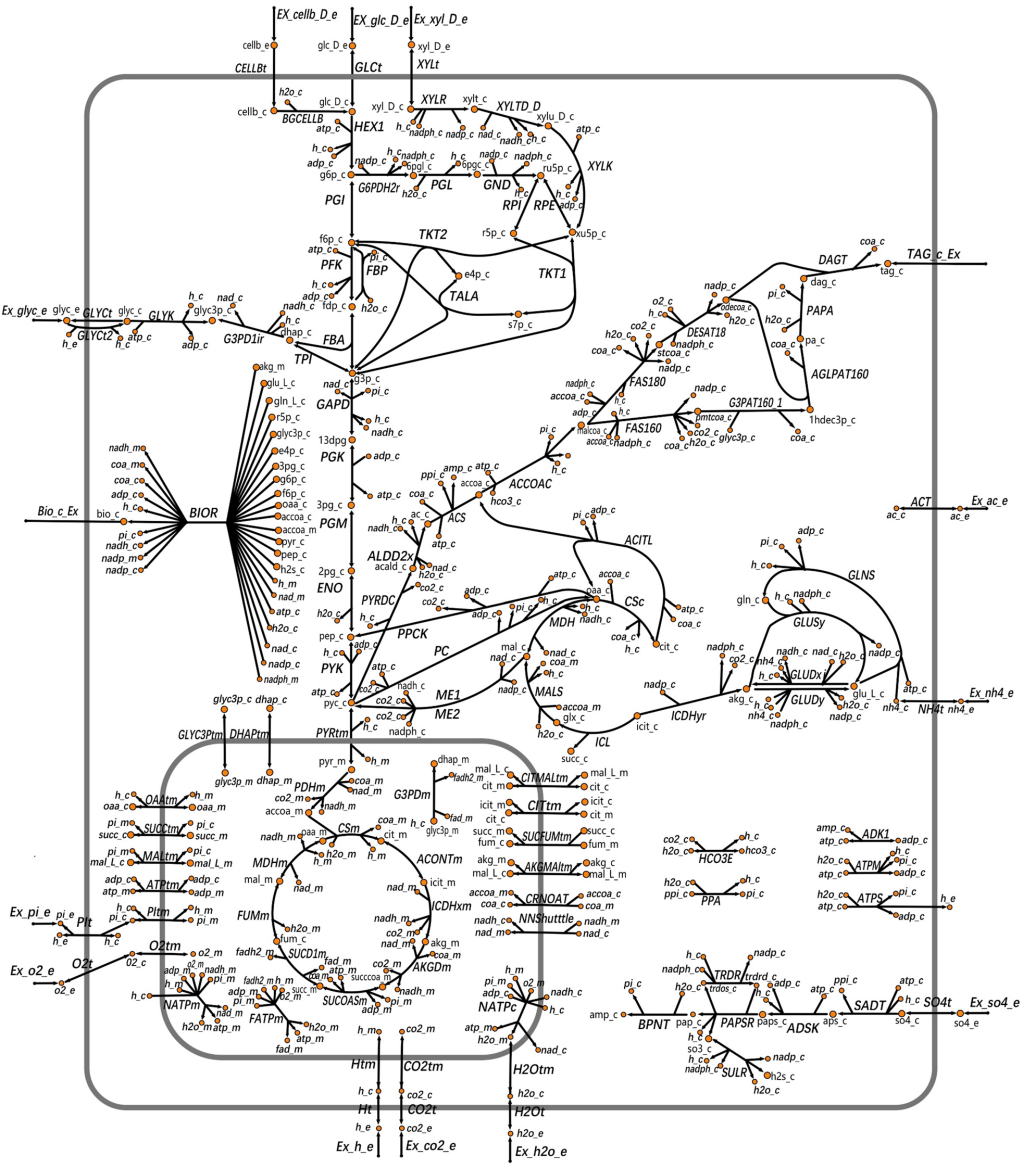
The visualization of a metabolic map of *L. starkeyi* NRRL Y-11557.  and  represent the metabolites involved in the reaction. The abbreviations with the e/c/m suffix are the shorter forms of the corresponding metabolites. The suffixes ‘_e’, ‘_c’ and ‘_m’ in the metabolite names refer to the ‘extracellular’, ‘cytosolic’ and ‘mitochondrial’ compartments, respectively. Black arrows represent the direction of reaction, one-way arrows represent the irreversible reaction, and the bidirectional arrows represent reversible reaction

All the reactions and metabolites in the small-scale metabolic model of *L. starkeyi* are summarized in the Additional file 1: Tables S1 and S2, respectively. The visualization of the metabolic map of *L. starkeyi* is depicted in Fig. 2. This model contained 112 metabolites, 123 reactions and 3 cell compartments including extracellular, cytoplasm, and mitochondria. The metabolic pathways included glycolysis, pentose phosphate pathway (PPP), tricarboxylic acid cycle (TCA), glyoxylate cycle, pyruvate dehydrogenase bypass, fatty acid (FA) synthesis pathway, and glycerolipid metabolism. The model involved in 13 exchange reactions and 31 transport reactions. The biomass reaction was used to analyze whether the metabolic model could normally generate biomass using a specific substrate. The model included the metabolism of 5 carbon sources including glucose, cellobiose, xylose, glycerol, and acetic acid. The P/O ratios of the mitochondrial NADH, mitochondrial FADH2, and cytoplasmic NADH were 2.5, 1.5, and 1.5, respectively.

### Prediction of lipid production potential from different carbon sources

**Fig. 3:**
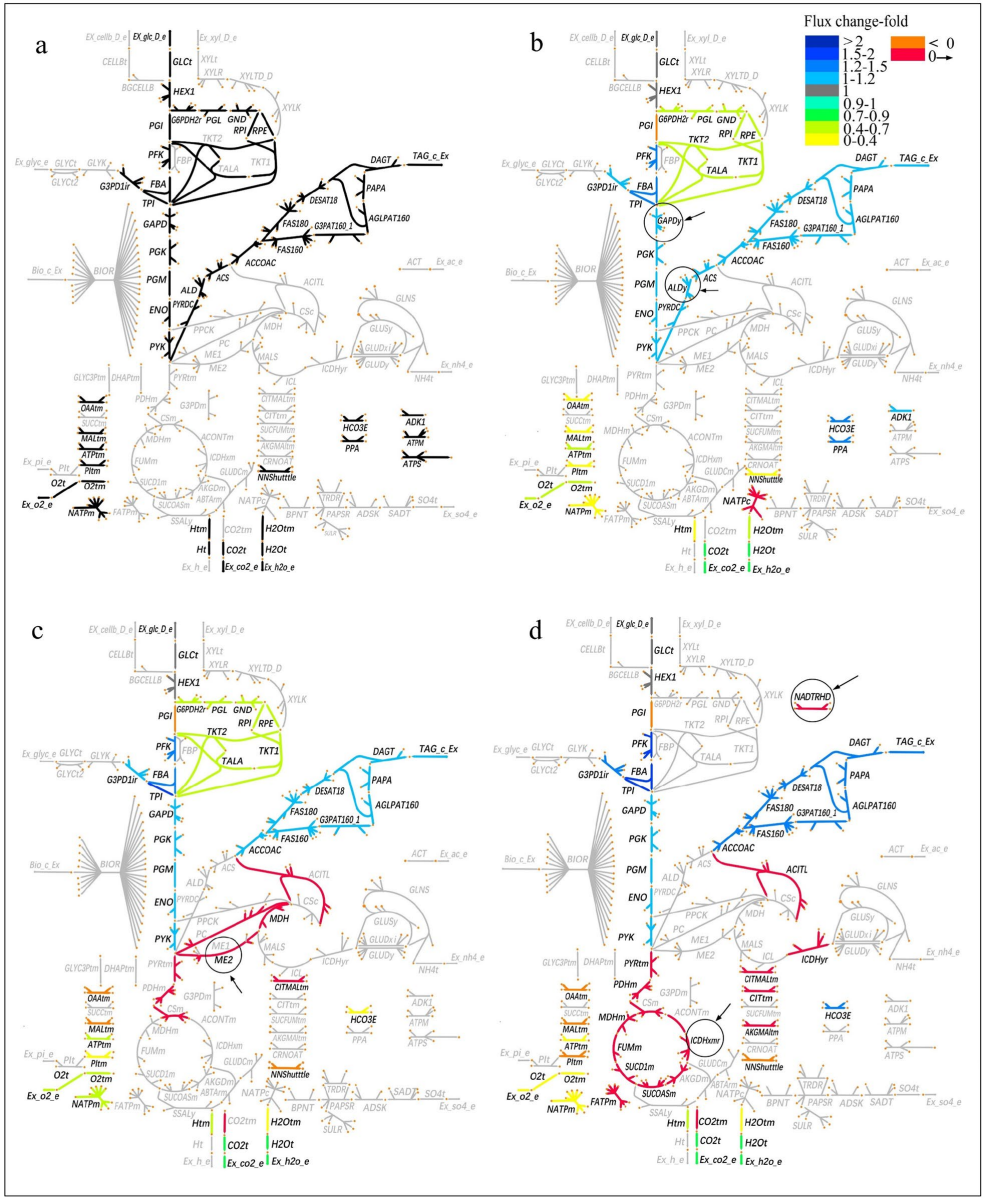
Optimum flux distribution of TAG synthesis from glucose under various modifications. **a** Original model, **b** GAPD or ALD was replaced by the NADP-dependent isoenzymes **c** addition of ME2, **d** addition of NADTRHD or ICDHxmr. Flux fold-change refers to the ratio of the flux of the modified model to the original model. Different colors indicate different flux change-fold. " < 0" means the reaction direction is opposite to the corresponding reaction in the original model, "0 → " means that the reaction with a flux of 0 in the original model has flux after modification. The relevant modifications are marked with circles

The theoretical lipid yields of glucose, cellobiose, xylose, glycerol, and acetic acid by *L. starkeyi* were calculated and the results are shown in Table 2. The optimal flux distribution for lipid production from glucose is shown in Fig. 3a. It was clear that the FA and triacylglycerol (TAG) synthesis pathways were very active, while the TCA cycle were severely stagnant (Fig. 3a). This phenomenon was in line with the transcriptome analysis result reported by Pomraning and co-workers [13].

**Table 2:** The theoretical lipid yield and related flux data of different carbon sources with various modifications by *L. starkeyi*

Target modification	Theoretical lipid yield		The source of NADH (mmol)			The source of NADPH (mmol)			Demand of ATP (mmol)	Acetyl-CoA production (mmol)	The carbon loss distribution (C-mol/C-mol, %)		
	(C-mol/C-mol)	(g/g)	Glycolysis	TCA	Others	PPP	NADH	Others			PPP	Glycolysis/ Gluconeogenesis	TCA
Glucose													
Original model	0.524	0.273	29.71	0	0	27.43	✗	0	63.62	14.86	22.86	24.76	0
NADP-dependent GAPDH	0.598	0.311	16.96	0	0	14.35	✗	16.96^d^	71.86	16.96	11.96	28.26	0
NADP-dependent ALD	0.598	0.311	16.96	0	0	14.35	✗	16.96^e^	71.86	16.96	11.96	28.26	0
NADP-dependent ME	0.602	0.313	34.15	0	0	13.62	17.89^a^	0	73.13	17.07	11.36	28.45	0
Transhydrogenases	0.643	0.335	38.60	2.11	0	0	32.62^b^	1.06^f^	59.71	18.24	0	32.16	3.52
Reversible ICDHxm	0.643	0.335	38.60	2.11	0	0	32.62^c^	1.06^f^	59.71	18.24	0	32.16	3.52
Cellobiose													
Original model	0.524	0.287	59.43	0	0	54.86	✗	0	121.90	29.71	22.86	24.76	0
NADP-dependent GAPDH	0.598	0.327	33.92	0	0	28.70	✗	33.91^d^	138.38	33.91	11.96	28.26	0
NADP-dependent ALD	0.598	0.327	33.92	0	0	28.70	✗	33.91^e^	138.38	33.91	11.96	28.26	0
NADP-dependent ME	0.606	0.332	68.73	0	0	25.88	37.56^a^	0	143.35	34.36	10.78	28.64	0
Transhydrogenases	0.649	0.355	77.17	3.59	0	0	66.13^b^	1.79^f^	114.67	36.79	0	32.15	2.99
Reversible ICDHxm	0.649	0.355	77.17	3.59	0	0	66.13^c^	1.79^f^	114.67	36.79	0	32.15	2.99
Xylose													
Original model	0.471	0.245	22.29	0	10^ g^	30.57	✗	0	49.05	11.14	30.57	22.29	0
NADP-dependent GAPDH	0.538	0.280	12.72	0	10^ g^	20.76	✗	12.72^d^	55.22	12.72	20.76	25.43	0
NADP-dependent ALD	0.538	0.280	12.72	0	10^ g^	20.76	✗	12.72^e^	55.22	12.72	20.76	25.43	0
NADP-dependent ME	0.583	0.303	27.56	0	10^ g^	14.15	21.29^a^	0	66.90	13.78	14.15	27.56	0
Transhydrogenases	0.641	0.334	32.17	1.87	10^ g^	0	37.03^b^	0.93^f^	50.55	15.15	0	32.17	3.74
Reversible ICDHxm	0.641	0.334	32.17	1.87	10^ g^	0	37.03^c^	0.93^f^	50.55	15.15	0	32.17	3.74
Glycerol													
Original model	0.524	0.267	14.86	0	0	13.71	✗	0	36.76	7.43	22.86	24.76	0
NADP-dependent GAPDH	0.598	0.304	8.48	0	0	7.17	✗	8.48^d^	39.79	8.48	11.96	28.26	0
NADP-dependent ALD	0.598	0.304	8.48	0	0	7.17	✗	8.48^e^	39.79	8.48	11.96	28.26	0
NADP-dependent ME	0.637	0.324	18.07	0	0	3.72	12.96^a^	0	45.31	9.03	6.20	30.11	0
Transhydrogenases	0.679	0.346	19.26	0	0	0	17.78^b^	0	37.41	9.63	0	32.10	0
Reversible ICDHxm	0.679	0.346	19.26	0	0	0	17.78^c^	0	37.41	9.63	0	32.10	0
Acetic acid													
Original model	0.470	0.245	0	9.27	0	4.49	✗	3.71^f^	31.11	4.45	11.24	4.60	37.14
NADP-dependent GAPDH	0.465	0.242	0	8.85	0	6.05	✗	3.25^f^	32.18	4.40	15.13	5.89	32.46
NADP-dependent ALD	0.470	0.245	0	9.27	0	4.49	✗	3.71^f^	31.11	4.45	11.24	4.60	37.14
NADP-dependent ME	0.470	0.245	0	9.27	0	4.49	0	3.71^f^	31.11	4.45	11.24	4.60	37.14
Transhydrogenases	0.485	0.252	0	10.48	0	0	3.40^b^	5.06^f^	29.74	4.58	0	0.88	50.63
Reversible ICDHxm	0.485	0.252	0	10.48	0	0	3.40^c^	5.06^f^	29.74	4.58	0	0.88	50.63

^a^The conversion of NADH to NADPH was mediated by NADP-dependent ME

^b, c^The conversion of NADH to NADPH was mediated by transhydrogenase and the reversible ICDHxm, respectively

^d, e^NADPH was generated from the reactions catalyzed by NADP-dependent GAPD and NADP-dependent ALD, respectively

^f^NADPH was generated from the reaction catalyzed by the cytoplasmic NADP-dependent ICDHyr

^g^NADH was generated from the reaction catalyzed by xylitol dehydrogenase

^✗^NADPH could not be derived from NADH

It should be noted that abundant NADPH is required for the FA synthesis. Generally, NADPH for FA synthesis is routinely supplied by the PPP and malic enzyme (ME) in oleaginous species [33]. However, NADPH originated from PPP meant carbon loss, which was undoubtedly detrimental to the lipid accumulation. Ratledge reported that the theoretical lipid yield of glucose was 0.316 g/g when both PPP and ME could serve as the suppliers of NADPH, while the yield was merely 0.276 g/g when PPP was the sole NADPH provider [33]. The ME in *L. starkeyi* AS 2.1560 was defined as NAD-dependent as described by Tang and co-workers [34]. The amino acid sequence of ME (ODQ72042) in *L. starkeyi* NRRL Y-11557 was virtually identical to that of *L. starkeyi* AS 2.1560. Thus, the ME in the model was recognized as NAD-dependent (ME1).

The theoretical lipid yields were merely 0.273, 0.287, 0.245, and 0.267, respectively, when glucose, cellobiose, xylose, or glycerol was the sole carbon source based on the model (Table 2). Among them, the yield on glucose was in agreement with the calculation result obtained by Ratledge [33]. The PPP was the unique supplier of NADPH. Indeed, the NADPH required for FA synthesis by *L. starkeyi* was majorly provided by PPP according to the C^13^ metabolic flux analysis using C^13^-labeled glycerol as substrate [14]. The NADH generated during the metabolic process from glucose to acetyl-CoA was excessive and could not be used for FA synthesis according to the flux distribution analysis. The redundant NADH was eventually channeled into oxidative phosphorylation to produce ATP, which led to an excess of ATP. On the other hand, glucose was partially shunted to PPP to produce NADPH as large amount of NADPH was required for the FA biosynthesis, which resulted in a lower theoretical lipid yield. Similar situations occurred when xylose, glycerol, and cellobiose were used as substrates. It was probably one of the major reasons why relatively low experimental lipid yields were obtained (Table 1).

The theoretical lipid yield was merely 0.245 g/g for acetic acid. Acetic acid was converted to acetyl-CoA by the acetyl-CoA synthase (ACS) before participating in cell metabolism [35, 36]. The acetylation of acetic acid needs the participation of ATP, resulting in a relatively high demand for ATP in acetic acid metabolism. As shown in Table 3, acetic acid was inferior to glucose for providing NADH and ATP, which led to more acetic acid consumption to meet the requirement. As shown in Table 2, up to 37.14% of acetic acid was consumed through TCA cycle. Therefore, acetic acid was not an excellent building block for lipid production.

**Table 3:** Maximal yields of ATP, NADH, and acetyl-CoA from glucose and acetic acid

Carbon source	ATP (mol/C-mol)	NADH (mol/C-mol)	Acetyl-CoA (mol/C-mol)
Glucose	5.33	1.96	0.33
Acetic acid	4.00	1.55	0.40

The lowest theoretical lipid yield was observed when xylose was used as substrate. It was because that xylose reductase needed one molecule of NADPH, which led to a greater demand for NADPH and more xylose channeling into PPP with an overall carbon loss of 30.57% (Table 2). This part of xylose was completely consumed to provide NADPH instead of conversion to acetyl-CoA as a precursor for FA synthesis. The more xylose was shunted to PPP, the less acetyl-CoA generated, resulting in a decrease in the theoretical lipid yield. Notably, when glucose, cellobiose, xylose, and glycerol were used as substrates, acetyl-CoA was always provided by pyruvate dehydrogenase bypass instead of ATP-citrate lyase (ACITL) to consume the redundant ATP as mentioned above.

### Prediction of potential targets for NADPH supply to enhance lipid production

As mentioned above, the redundancy of NADH is one of the major issues against the theoretical lipid yield. Converting the redundant NADH to NADPH has great potential to reduce the carbon loss and enhance the theoretical lipid yield. Here, some modifications for enriching the supply of NADPH were evaluated in the metabolic model and the results are summarized in Table 2.

When glucose was used as substrate, the NADH generation was majorly referring to glyceraldehyde-3-phosphate dehydrogenase (GAPD) and aldehyde dehydrogenase (ALD) (Fig. 3a). The theoretical lipid yield was analyzed when either GAPD or ALD in the model was replaced by the NADP-dependent isoenzymes (GAPDy and ALDy). Specifically, the flux for glycolysis and FA biosynthesis were significantly increased when glucose was used as substrate (Fig. 3b vs Fig. 3a). The reaction catalyzed by GAPDy or ALDy could provide 54.17% of the required NADPH, which resulted in the proportion of glucose flowing to PPP decreasing from 22.86% to 11.96% and a significant decline of carbon loss (Table 2 and Fig. 3b). The theoretical lipid yield increased significantly from 0.273 g/g to 0.311 g/g. However, 20.76% of xylose remained channeling into PPP despite the modification, leading to a significantly lower lipid yield of 0.280 g/g.

NADH can be changed to NADPH through a transhydrogenase cycle involving in pyruvate decarboxylase, malate dehydrogenase and NADP-dependent malic enzyme (ME2) [33]. Thus, ME2 was introduced into the metabolic model and the theoretical lipid yield was recalculated. The theoretical lipid yields of glucose, cellobiose, xylose, and glycerol, increased by 14.7%, 15.7%, 23.7%, and 21.3%, respectively (Table 2). As shown in Table 2, 56.78% of the required NADPH was provided by ME2 when glucose was used as substrate. Similarly, the flux for glycolysis and FA biosynthesis were significantly increased with a sharp decline of the flux of PPP (Fig. 3c vs Fig. 3a). Specifically, the proportion of glucose channeling into PPP decreased from 22.86% to 11.36%, and the theoretical lipid yield increased from 0.273 g/g to 0.313 g/g concurrently (Table 2). It was worth noting that when glycerol was used as the substrate, the enhancement in the theoretical lipid yield was the most significant. The proportion of glycerol flowing to PPP decreased from 22.86% to 6.20%, and theoretical lipid yield increased from 0.267 g/g to 0.324 g/g. It was because extra FADH2 was produced during the metabolism from glycerol to acetyl-CoA, which meant that more NADPH could be produced through ME2. The overexpression of ME2 in oleaginous fungus *Mucor circinelloides* resulted in a 2.5-fold increase in lipid content [37]. When the ME2 gene originated from *M. circinelloides* was expressed into *Rhodotorula glutinis*, the lipid content was increased significantly from 19 to 39% [38]. It was indicated that when ME2 can be introduced into *L. starkeyi*, it should have positive effect on lipid production.

The ME2-mediated NADPH production necessitates the consumption of ATP, which limits the theoretical lipid yield to a certain extent (Table 2). Here, NAD-dependent transhydrogenase (NADTRHD), changing NADH to NADPH directly without the necessity of ATP, was introduced into the metabolic model. An alternative approach to provide NADPH is reversing the direction of the mitochondrial NAD-dependent isocitrate dehydrogenase (ICDHxm) proposed by Ratledge [33]. NADH can be changed to NADPH without ATP consumption by a reversible NAD-dependent isocitrate dehydrogenase (ICDHxmr) and cytoplasmic NADP-dependent isocitrate dehydrogenase (ICDHyr). The introduction of ICDHxmr or NADTRHD has identical positive effect and the theoretical lipid yield of glucose reached as high as 0.335 g/g (Table 2). The optimum flux distribution for TAG production is depicted in Fig. 3d. It was clear that no glucose was channeled into PPP, indicating that NADPH was no longer provided by PPP. Instead, part of acetyl-CoA was channeled into TCA cycle to provide NADH changing to NADPH (Fig. 3d). The NADPH required for lipid synthesis was predominantly derived from NADH. Interestingly, when glycerol was used as sole carbon source, the theoretical lipid yield reached a higher value of 0.346 g/g. It was because that the NADH generated from glycerol to acetyl-CoA metabolism was sufficient. Acetyl-CoA was not necessary to be consumed to provide NADH. The carbon loss was merely involved in glycolysis (Table 2). Interestingly, the theoretical lipid yield of xylose was significantly increased to 0.334 g/g after the modifications, which was comparable to that of glucose (Table 2). The NADPH required by xylose reductase could be provided by NADH generated by xylitol dehydrogenase by the modifications.

It should be noted that all the modifications mentioned above exerted no significantly positive effects on the theoretical lipid yield when acetic acid was used as substrate. There was no redundant NADH because the metabolic process from acetic acid to acetyl-CoA did not produce NADH concurrently. Moreover, acetic acid was acetylated by ACS without involving in glycolysis and pyruvate dehydrogenase bypass pathways. Thus, ME2, GAPDy or ALDy not produce a marked effect on the theoretical lipid yield. On the other hand, the metabolic process from acetyl-CoA to PPP underwent gluconeogenesis, which resulting in greater carbon loss. The introduction of NADTRHD or ICDHxmr could slightly increase the theoretical lipid yield of acetic acid, as acetyl-CoA can be channeled into TCA cycle to produce NADH.

### Prediction of lipid production by combined utilization of different carbon sources

Glucose and xylose are the two major monomeric sugars in the lignocellulosic hydrolysates. In addition, cellobiose and acetic acid are routinely co-present. Interestingly, combined utilization of carbon sources mixtures has been widely investigated for lipid production by *L. starkeyi* [4, 5, 26, 29, 39–42]. Most microorganisms prefer glucose over other monomeric sugars due to glucose catabolite repression. Sequential consumption of mixed carbon sources will prohibit the optimization of metabolic flux to give the yield advantage. It was worth mentioning that some mixed carbon sources including xylose/acetic acid [4], glucose/xylose/acetic acid [5, 39], cellobiose/xylose [40], and glucose/mannose [42] could be assimilated simultaneously by *L. starkeyi*. In addition, some feasible strategies including genetic modification and directed evolution have been developed to circumvent the catabolite repression and realize simultaneously utilize of mixed carbon sources [43–45]. Interestingly, simultaneous utilization of glucose and xylose was also realized by carefully controlling glucose concentration and dilution rate in chemostat culture [46]. A variety of strategies can be selected to circumvent sequential utilization issues when model-verified substrates combination shows benefit for lipid biosynthesis. Thus, it is meaningful to assess the potential of combined utilization of different low-cost carbon sources for improving the lipid yield. Here, the combined utilization of different carbon sources was analyzed and the theoretical lipid yields were calculated based on the model. Glucose, glycerol, xylose, and cellobiose mixed in pairs could not significantly improve the theoretical lipid yield (data not shown). In addition, the theoretical lipid yields were virtually identical regardless of the mass ratios of xylose and cellobiose, which was in accordance with the results reported by Gong and co-workers [40].

**Fig. 4:**
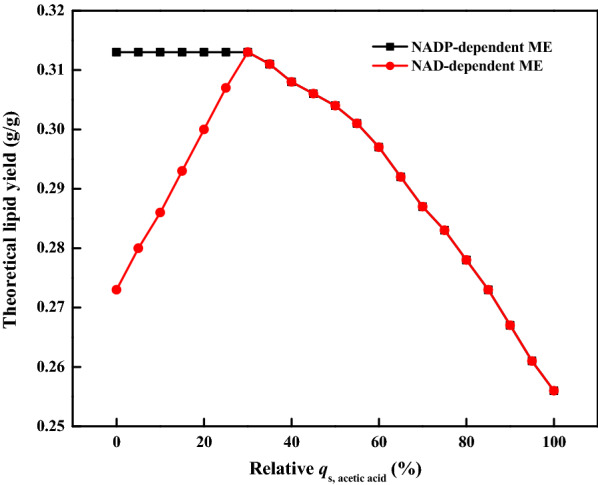
The effect of the acetic acid and glucose co-utilization strategy on the theoretical lipid yield. The black and red lines represent NADP-dependent ME and NAD-dependent ME, respectively

Interestingly, the theoretical lipid yield could be significantly improved when acetic acid was co-present with other carbon sources including glucose, cellobiose, xylose, or glycerol (Table 4). Among them, the combined utilization of acetic acid and glycerol could achieve the highest theoretical lipid yield (C-mol/C-mol). It was because one more molecule of FADH2 could be produced in the metabolism of glycerol to acetyl-CoA compared with sugars including glucose, cellobiose, and xylose. For acetic acid/glucose mixture, the effect of the acetic acid proportion on the theoretical lipid yield was investigated and the results are shown in Fig. 4. The theoretical lipid yield was continuously increased as the increase of the relative *q*_s, acetic acid_ from zero to 30% (Table 5 and Fig. 4). The highest value reached 0.313 g/g when the relative *q*_s, acetic acid_ reached 30%. In this case, acetic acid was totally channeled into acetyl-CoA as precursor for FA synthesis and the NADH produced from the metabolism of glucose into acetyl-CoA met the requirement. The acetylation of acetic acid requires the consumption of ATP. The ATP was provided by the oxidative phosphorylation process, which was beneficial for alleviating the wastage of the excessive NADH. As shown in Table 5, the proportion of glucose flowing to PPP increased as the increase of acetic acid flux. The increase in the theoretical lipid yield at this stage was majorly due to the consumption of the redundant NADH via acetic acid metabolism and the increment of acetyl-CoA for FA synthesis.

**Table 4:** Prediction of the theoretical lipid yield by combined utilization of acetic acid and other carbon sources

Carbon source	Relative *q*_s, acetic acid_ (%)	Theoretical lipid yield (C-mol/C-mol)	Theoretical lipid yield (g/g)
Acetic acid/glucose	30	0.602	0.313
Acetic acid/cellobiose	35	0.593	0.325
Acetic acid/xylose	40	0.585	0.304
Acetic acid/glycerol	35	0.620	0.316

**Table 5:** Prediction of the theoretical lipid yield and related flux data by combined utilization of acetic acid and glucose

Relative *q*_s, acetic acid_ (%)	Theoretical lipid yield (g/g)	Demand of ATP (mmol)	Acetyl-CoA production (mmol)	The proportion of the flux distribution of glucose (%)			The proportion of the flux distribution of acetic acid (%)		
				PPP	Lipid precursors^a^	TCA	PPP	Lipid precursors	TCA
0	0.273	63.62	14.86	22.86	77.14	0	–	–	–
15	0.293	63.49	15.97	28.91	71.09	0	0	100	0
30	0.313	55.25	17.10	37.48	62.32	0.21	0	100	0
50	0.304	56.95	16.53	45.36	21.65	32.99	0	100	0
70	0.287	64.18	15.65	60.53	10.04	29.43	0	74.55	25.45
90	0.267	75.09	14.55	100	0	0	6.54	58.04	35.42
100	0.256	84.05	13.92	–	–	–	17.79	49.95	32.25

^a^: Lipid precursors were consisted of acetyl-CoA and glycerol

The theoretical lipid yield was continuously decreased when the acetic acid proportion was exceeding 30% (Fig. 4). The demand for ATP increased with the increase of acetic acid flux. The NADH generated via the metabolism of glucose to acetyl-CoA became inadequate. Acetyl-CoA began to be consumed through TCA cycle to provide NADH, which led to a continuously increase in carbon loss and decrease in theoretical lipid yield (Table 5). When the relative *q*_s, acetic acid_ exceeded 85.1%, glucose was totally channeled into PPP to supply NADPH. However, the NADPH remained inadequate to support the FA synthesis. Part of the acetyl-CoA was channeled into gluconeogenesis and PPP to produce NADPH.

The metabolic flux and theoretical lipid yield were analyzed when ME2 was added into the model. As depicted in Fig. 4, the theoretical lipid yield could be maintained at a relatively high level when the relative *q*_s, acetic acid_ was lower than 30%. Interestingly, the yields became identical when the relative *q*_s, acetic acid_ was higher than 30% regardless of the presence of ME2. In the presence of ME2, the transhydrogenase cycle could change the redundant NADH to NADPH for the FA synthesis, which led to the decrease in glucose channeling into PPP and the increase in lipid yield. There was redundant NADH in the metabolic network when the relative *q*_s, acetic acid_ was lower than 30%. This part of NADH could be converted into NADPH through ME2, which reduced the flux of glucose flowing to PPP. The redundant NADH in the metabolic network gradually decreased as the acetic acid flux increased. Correspondingly, the flux of ME2 gradually decreased. The ME2 did not work when relative *q*_s, acetic acid_ was higher than 30% because there was no redundant NADH in the metabolic network.

Acetic acid was not an excellent carbon source when it was used as the sole carbon source. Interestingly, when acetic acid was used in combination with other carbon sources including glycerol, glucose, cellobiose, and xylose, the lipid production could be improved. Actually, acetic acid and sugars co-fermentation has been validated as a promising strategy for lipid production by some oleaginous species [36, 47, 48]. Acetic acid was reported to be assimilated simultaneously with glucose and xylose by *L. starkeyi* [4, 5, 39]. The *q*_s, acetic acid_ was reported slightly lower than the *q*_s, glucose_ [5]. Acetic acid has been considered as a sustainable and low-cost substrate from a wide array of sources [49]. Especially, acetic acid is routinely co-present with lignocellulosic hydrolysates and can reach concentrations as high as 10 g/L depending on the feedstock and the pretreatment method [4, 49]. Thus, the combination utilization strategy will probably be a very promising strategy for lipid production.

### Prediction of lipid production by the RBO pathway

**Fig. 5:**
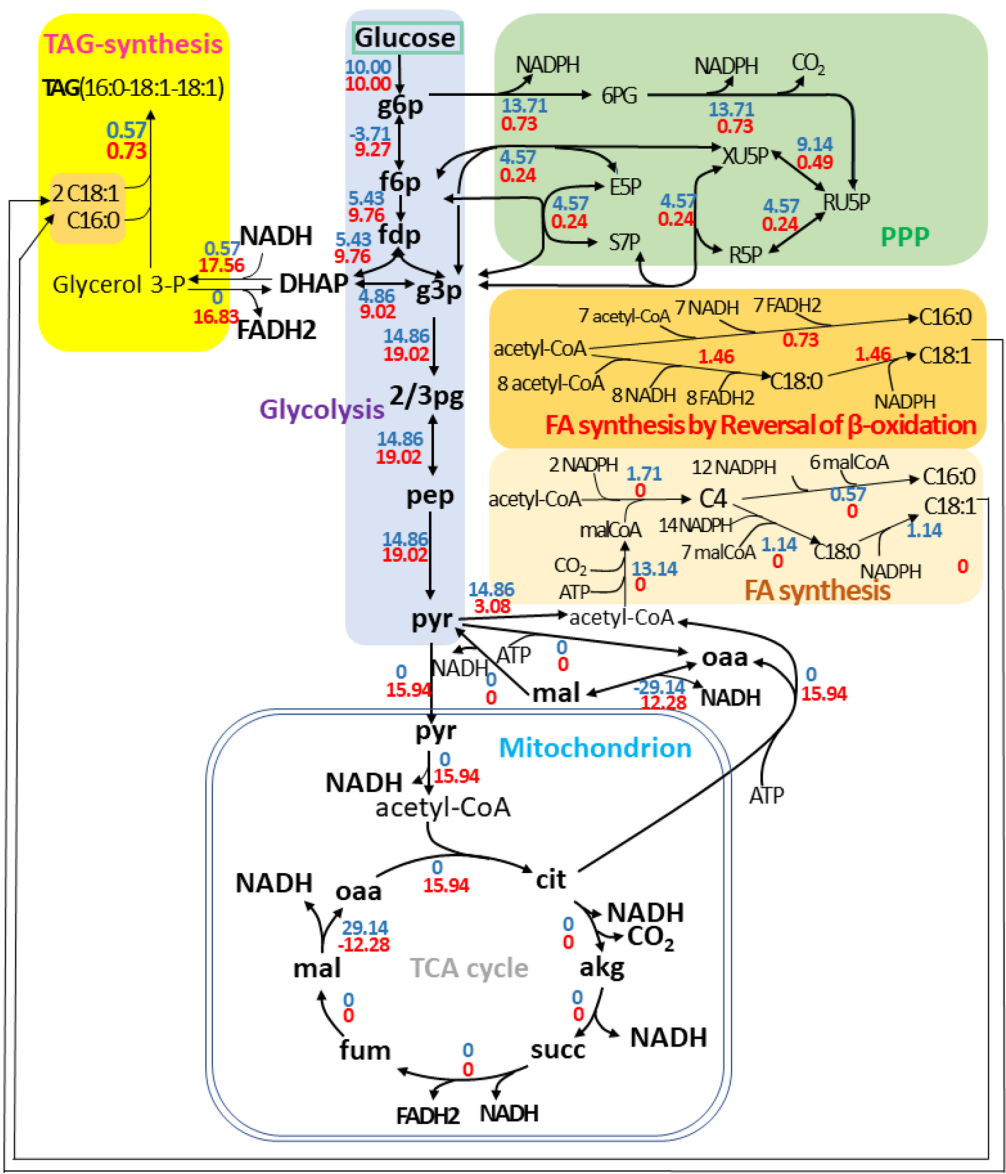
Optimum flux distribution of TAG synthesis from glucose. The blue and red numbers represent the optimal flux distributions through the FA synthesis pathway and the RBO pathway, respectively

Dellomonaco and co-workers proposed a novel RBO pathway to synthesize alcohols or carboxylic acids with various chain lengths [50]. This pathway can save large amount of ATP as the conversion of acetyl-CoA to malonyl-CoA was not a necessity. In addition, NADH is the majorly required coenzyme in the RBO route. There was no need to change the redundant NADH to NADPH for FA synthesis. Here, a hypothetical RBO pathway was introduced into the metabolic model to produce long-chain FA (C16:0 and C18:1). The hypothetical reactions (FAS160* and FAS180*) are described in Additional file 1: Table S1. The theoretical lipid yields of glucose, cellobiose, xylose, glycerol, and acetic acid were all significantly increased when the RBO pathway was introduced (Table 6). Interestingly, the theoretical lipid yields of glucose, cellobiose, and acetic acid were all achieved the maximal improvement among all the modification strategies (Table 6 vs Table 2). The effect of the RBO pathway on the optimal flux distribution of glucose is illustrated in Fig. 5. The FA was no longer produced through the FA synthesis pathway but through the RBO pathway when this pathway was introduced (Fig. 5). In addition, part of the acetyl-CoA was provided by pyruvate dehydrogenase bypass. The amount of ATP for lipid synthesis was reduced, and the NADH generated in the process from glucose to acetyl-CoA was sufficient to meet the requirement for lipid synthesis. Acetyl-CoA was no longer consumed through TCA cycle to provide NADH. The maximal lipid yield of glucose should be 0.679 C-mol/C-mol only taking the minimal carbon loss into account using TAG (16:0,18:1,18:1) as the target product. As depicted in Table 6, the theoretical lipid yields of glucose, cellobiose, and glycerol were infinitely approaching this value, indicating the great potential of the RBO pathway for the FA production. It was worth noting that the enhancement was majorly due to the reduction of ATP requirement in the process of lipid synthesis. The theoretical lipid yield of xylose was merely 0.314 g/g, which was significantly lower than that of the modification of NADTRHD or ICDHxmr. It was because the RBO pathway caused severer carbon loss through PPP as the NADPH required for xylose metabolism could not be transformed from NADH (Table 6 vs Table 2).

**Table 6:** Prediction of the theoretical lipid yield and related flux data of different carbon sources through the RBO pathway

Carbon source	Theoretical lipid yield		The source of NADH (mmol)			The source of FADH2 (mmol)		The source of NADPH (mmol)		Demand of ATP (mmol)	Acetyl-CoA production (mmol)	The carbon loss distribution (C-mol/C-mol, %)		
	(C-mol/C-mol)	(g/g)	Glycolysis	TCA	Other	G3PDm^a^	TCA	PPP	ICDHyr^b^			PPP	Pyruvate metabolism	TCA
Glucose	0.671	0.349	38.05	0	0	16.83	0	1.46	0	47.20	19.02	1.22	31.70	0
Cellobiose	0.671	0.367	76.10	0	0	33.66	0	2.92	0	94.39	38.04	1.22	31.70	0
Xylose	0.604	0.314	28.54	0	10^c^	12.62	0	11.10	0	47.65	14.27	11.10	28.54	0
Glycerol	0.671	0.342	19.02	0	0	9.63	0	0.73	0	34.36	9.51	1.22	31.70	0
Acetic acid	0.544	0.283	0	13.78	0	0	4.66	0	0.40	25.73	5.14	0	0.99	44.62

^a^FADH2 was generated from the reaction catalyzed by the mitochondrial glycerol-3-phosphate dehydrogenase

^b^NADPH was generated from the reaction catalyzed by the cytoplasmic NADP-dependent ICDHyr

^c^NADH was generated from the reaction catalyzed by xylitol dehydrogenase

At present, most of the experiments referring to the RBO pathway were carried out in prokaryotic cell such as *Escherichia coli*. The RBO pathway is in favor of short and medium-chain FA production because it is easy to control product lengths [50–52]. For example, Dellomonaco et al. reported that the production of n-butanol by *E. coli* through the RBO strategy reached 14 g/L [50]. Recently, the production of medium-chain FA by *E. coli* via the RBO pathway reached 3.8 g/L [52]. It was worth mentioning that the introduction of RBO pathway was successful in the eukaryote *Saccharomyces cerevisiae.* Lian et al. reported the production of 20 mg/L n-butanol through this strategy [53]. There was no report involving in the RBO strategy in the oleaginous species. The implementation of this novel strategy to improve the lipid yield should be a challenging task.

## Conclusions

A small-scale metabolic model of *L. starkeyi* was successfully constructed for predicting the lipogenesis potential from diverse low-cost substrates. The theoretical lipid yields were relatively low according to the metabolic model. The redundant NADH generated could not be directly used for the synthesis of FA, while part of the carbon source was shunted to PPP to provide NADPH, which caused the decline in theoretical lipid yield. Some modifications were proposed in the model to effectively use the redundant NADH, reduce the carbon loss, and increase the theoretical lipid yield. Combined utilization of acetic acid and sugars/glycerol was a promising strategy to enhance the lipid accumulation potential. The theoretical lipid yield could be significantly improved through the RBO pathway, suggesting the great potential of this strategy to overproduce long-chain FA. In future, these strategies should be experimentally verified and a genome-scale metabolic model of *L. starkeyi* should be constructed for more accurate and comprehensive prediction.

## Methods

### Strain

*L. starkeyi* NRRL Y-11557 was used throughout the study. The complete genome sequence was published in 2016, and the GenBank entry number was GCA_001661325.1 [54]. The genome annotation information can be found on JGI MycoCosm. *L. starkeyi* NRRL Y-11557 is also numbered as NRRL Y-1388, ATCC 58,680, CBS 1807, DSM 70,925, IFO 1289, JCM 5995, etc., in different preservation centers [1].

### Construction of metabolic model

Genome-scale metabolism models of *i*MM904 and yeast 8.3.5, as well as the small-scale metabolism models of *R. toruloides* and *C. oleaginosum*, were used as reference models for the construction of the small-scale metabolism model of *L. starkeyi*. All metabolites and reactions in the model refer to KEGG, BIGG and yeast 8.3.5. Subcellular localization of reactions referred to CELLO2GO and the visualization of metabolic model referred to Escher.

The gene information related to glycolysis, pyruvate metabolism, tricarboxylic acid cycle (TCA), pentose phosphate pathway (PPP), glyoxylate cycle, oxidative phosphorylation, fatty acid (FA) synthesis, and glycerolipid metabolism were obtained from JGI MycoCosm. Oleic acid (C18:1) and palmitic acid (C16:0) are routinely the predominant components in the fatty acid composition of *L. starkeyi* [4]. Therefore, TAG (16:0,18:1,18:1) was set as the target in the metabolic model. Since the biomass composition of *L. starkeyi* NRRL Y-11557 was not determined, the biomass response equation was quote directly [22, 23, 55, 56]. The metabolic reactions of ammonia nitrogen, sulfate, glucose, cellobiose, xylose, glycerol, and acetic acid were added to the rough model. The exchange reactions and the transport reactions between mitochondria and cytoplasm were also added. The transport reactions refer to the genome annotation of *L. starkeyi*, yeast 8.3.5, *i*MM904, and the small-scale metabolic models of *R. toruloides* and *C. oleaginosum* [22, 23]. Through the system feasibility test, the transport reaction of corresponding metabolite was supplemented to fill the gap based on the blocked reaction. In addition, the spontaneous reactions and ATP maintenance reactions (ATPM) were supplemented to the rough model.

The non-growth associated maintenance (NGAM) was expressed by the ATPM, pseudo-reaction consuming ATP and representing wasted energy [22]. The theoretical lipid yield should be overestimated when the minimal flux of ATPM was not restricted. Thus, NGAM in the model was represented by setting the lower limit of ATPM to a minimum value based on a published model [22]. The minimal flux for ATPM was determined according to the following equation:

1 $${V}_{NGAM}={m}_{S}\times {y}_{ATP/S},$$

where *m*_*S*_ is the maintenance coefficient, and the *y*_*ATP/S*_ is the theoretical maximal ATP yield based on a specific substrate. *y*_*ATP/S*_ was calculated by setting ATPM as the objective function based on the model. The *m*_*S*_ coefficient of *L. starkeyi* was 30 mg xylose/g cell mass/h according to the estimation by Anschau and co-workers [57].

### Model calibration

The analysis of the model was carried out with the help of CellNetAnalyzer running on the MATLAB R2013a platform [58]. The system feasibility and redundancy were tested for the preliminary version of the metabolic network. If the results showed that the operation failed, searching the blocked reactions. Pay attention to the transport reaction between mitochondria and cytoplasm. If it remained unresolved, the entire metabolic model should be divided into several parts by adding the output of some metabolites. The flux optimization analysis was conducted to check if the system could be operated normally. The input flux of glucose, cellobiose, xylose, glycerol, or acetic acid was set at 10 mmol/g/h. FBA and flux optimization analysis were conducted to check if biomass and TAG could be synthesized normally.

### In silico analysis of the metabolic network

The coefficients of the biomass reaction and the TAG exchange reaction were set at 0 and -1, respectively, to ensure that the flux of TAG was maximized when the FBA was performed. To calculate the theoretical lipid yield of glucose, cellobiose, xylose, glycerol or acetic acid, the input flux of the specific carbon source was set at 10 mmol/g/h. The actual absorption rate of each carbon source was not considered. For the combination utilization of acetic acid and glucose, the total specific uptake rates (*q*_s, global_) were always set to 1.8 g_substrate_/g/h, corresponding to 10 mmol_glucose_/g/h. The relative specific acetic acid uptake rate (*q*_s, acetic acid_) was expressed as the proportion of *q*_s, acetic acid_ accounting for the *q*_s, global_. The effect of relative *q*_s, acetic acid_ ranging from zero to 100% on the yield advantage was estimated. The FBA was performed to obtain the optimal metabolic flux distribution for the synthesis of TAG. After conversion, the theoretical lipid yield corresponding to the input carbon source was obtained.

## Supplementary Information

**Additional file 1: Table S1.** Reactions in the small-scale metabolic model of *L. starkeyi* NRRL Y-11557. **Table S2.** Metabolites in the small-scale metabolic model of *L. starkeyi* NRRL Y-11557.

## Data Availability

All appropriate data for this study has been included in the manuscript.

## Abbreviations

FA: Fatty acid
RBO: Reverse β-oxidation
PPP: Pentose phosphate pathway
FBA: Flux balance analysis
NRRL: Agricultural Research Service Culture Collection
TAG: Triacylglycerols
TCA: Tricarboxylic acid
ME: Malic enzyme
ME1: NAD-dependent malic enzyme
ME2: NADP-dependent malic enzyme
ACITL: ATP-citrate lyase
GAPD: Glyceraldehyde-3-phosphate dehydrogenase
GAPDy: NADP-dependent glyceraldehyde-3-phosphate dehydrogenase
ALD: Aldehyde dehydrogenase
ALDy: NADP-dependent aldehyde dehydrogenase
NADTRHD: NAD-dependent transhydrogenase
ICDHyr: NADP-dependent isocitrate dehydrogenase
ICDHxm: Mitochondrial NAD-dependent isocitrate dehydrogenase
ICDHxmr: Reversible NAD-dependent isocitrate dehydrogenase
FAS 160*: Fatty acid (16:0) synthesis reaction through RBO pathway
FAS 180*: Fatty acid (18:0) synthesis reaction through RBO pathway
ATPM: ATP maintenance reactions
NGAM: Non-growth associated maintenance
