# Supplementary material for: A metabolic model of Lipomyces starkeyi for predicting lipogenesis potential from diverse low-cost substrates
Source: Biotechnol Biofuels. 2021 Jul 1;14:148. doi: 10.1186/s13068-021-01997-9 (PMC8247262; doi:10.1186/s13068-021-01997-9)
Supplement: Supplementary file 1 — Additional file 1: Table S1. Reactions in the small-scale metabolic model of L. starkeyi NRRL Y-11557. Table S2. Metabolites in the small-scale metabolic model of L. starkeyi NRRL Y-11557. [file 13068_2021_1997_MOESM1_ESM.docx]

Table S1 Reactions in the small-scale metabolic model of *L. starkeyi* NRRL Y-11557

| Abbreviation | Name | Equation | EC-Number | JGI MycoCosm protein ID | Subsystem |
| --- | --- | --- | --- | --- | --- |
| HEX1 | Hexokinase | atp_c + glc_D_c → adp_c + g6p_c + h_c | 2.7.1.1, 2.7.1.2 | 3313, 74883, 89420 | Glycolysis |
| PGI | Glucose-6-phosphate isomerase | g6p_c ⇌ f6p_c | 5.3.1.9 | 1064 | Glycolysis / Gluconeogenesis |
| PFK | Phosphofructokinase | atp_c + f6p_c → adp_c + fdp_c + h_c | 2.7.1.11 | 59861 | Glycolysis |
| FBP | Fructose-bisphosphatase | fdp_c + h2o_c → f6p_c + pi_c | 3.1.3.11 | 324941 | Gluconeogenesis |
| FBA | Fructose-bisphosphate aldolase | fdp_c ⇌ dhap_c + g3p_c | 4.1.2.13 | 72319 | Glycolysis / Gluconeogenesis |
| TPI | Triose-phosphate isomerase | dhap_c ⇌ g3p_c | 5.3.1.1 | 201989, 196787, 197367 | Glycolysis / Gluconeogenesis |
| GAPD | Glyceraldehyde-3-phosphate dehydrogenase | g3p_c + nad_c + pi_c ⇌ 13dpg_c + h_c + nadh_c | 1.2.1.12 | 68472 | Glycolysis / Gluconeogenesis |
| G3PD1ir | Glycerol 3 phosphate dehydrogenase | dhap_c + h_c + nadh_c → glyc3p_c + nad_c | 1.1.1.8 | 50182 | Glycerophospholipid metabolism |
| PGK | Phosphoglycerate kinase | 3pg_c + atp_c ⇌ 13dpg_c + adp_c | 2.7.2.3 | 75322 | Glycolysis / Gluconeogenesis |
| PGM | Phosphoglycerate mutase | 2pg_c ⇌ 3pg_c | 5.4.2.11 | 69043, 2139 | Glycolysis / Gluconeogenesis |
| ENO | Enolase | 2pg_c ⇌ h2o_c + pep_c | 4.2.1.11 | 2413 | Glycolysis / Gluconeogenesis |
| PYK | Pyruvate kinase | adp_c + h_c + pep_c → atp_c + pyr_c | 2.7.1.40 | 215 | Glycolysis |
| PC | Pyruvate carboxylase |  | 6.4.1.1 | 70534, 97474 | TCA cycle |
| PPCK | Phosphoenolpyruvate carboxylase | atp_c + oaa_c → adp_c + co2_c+ pep_c | 4.1.1.49 | 934 | Gluconeogenesis |
| G6PDH2r | Glucose-6-phosphate dehydrogenase | g6p_c + nadp_c → 6pgl_c + h_c + nadph_c | 1.1.1.49 | 75163 | Pentose phosphate pathway |
| PGL | 6-phosphogluconolactonase | 6pgl_c + h2o_c → 6pgc_c + h_c | 3.1.1.31 | 5857 | Pentose phosphate pathway |
| GND | Phosphogluconate dehydrogenase | 6pgc_c + nadp_c → co2_c + nadph_c + ru5p_D_c | 1.1.1.44 | 1792 | Pentose phosphate pathway |
| RPI | Ribose-5-phosphate isomerase | r5p_c ⇌ ru5p_D_c | 5.3.1.6 | 62277, 244782 | Pentose phosphate pathway |
| RPE | Ribulose-5-phosphate epimerase | ru5p_D_c ⇌ xu5p_D_c | 5.1.3.1 | 87988 | Pentose phosphate pathway |
| TKT1 | Transketolase | r5p_c + xu5p_D_c ⇌ g3p_c + s7p_c | 2.2.1.1 | 73561, 3128, 337764 | Pentose phosphate pathway |
| TKT2 | Transketolase | e4p_c + xu5p_D_c ⇌ f6p_c + g3p_c | 2.2.1.1 | 73561, 3128, 337765 | Pentose phosphate pathway |
| TALA | Transaldolase | g3p_c + s7p_c ⇌ e4p_c + f6p_c | 2.2.1.2 | 1896, 7020 | Pentose phosphate pathway |
| PDHm | Pyruvate dehydrogenase | coa_m + nad_m + pyr_m → accoa_m + co2_m + nadh_m | 1.2.4.1, 1.8.1.4, 2.3.1.12 | 74867, 1724, 79742,6742 | Glycolysis |
| CSm | Citrate synthase | accoa_m + h2o_m + oaa_m → cit_m + coa_m + h_m | 2.3.3.1 | 68065, 92627 | TCA cycle |
| ACONTm | Aconitate hydratase | cit_m ⇌ icit_m | 4.2.1.3 | 3917, 72571 | TCA cycle |
| ICDHxm | Isocitrate dehydrogenase | icit_m + nad_m → akg_m + co2_m + nadh_m | 1.1.1.41 | 4980, 70496 | TCA cycle |
| ICDHyr | Isocitrate dehydrogenase (NADP) | icit_c + nadp_c → akg_c + co2_c + nadph_c | 1.1.1.42 | 85639 | TCA cycle |
| AKGDm | 2-oxoglutarate dehydrogenase | akg_m + coa_m + nad_m → co2_m + nadh_m + succoa_m | 1.2.4.2, 2.3.1.61, 1.8.1.4 | 246003, 3320, 67480 | TCA cycle |
| SUCOASm | Succinate CoA ligase ADP forming | atp_m + coa_m + succ_m ⇌ adp_m + pi_m + succoa_m | 6.2.1.5 | 60710, 2214 | TCA cycle |
| SUCD1m | Succinate dehydrogenase | fad_m + succ_m ⇌ fadh2_m + fum_m | 1.3.5.1 | 28592, 58405 | TCA cycle |
| FUMm | Fumarase mitochondrial | fum_m + h2o_m ⇌ mal_L_m | 4.2.1.2 | 60314 | TCA cycle |
| MDHm | Malate dehydrogenase mitochondrial | mal_L_m + nad_m ⇌ h_m + nadh_m + oaa_m | 1.1.1.37 | 5229 | TCA cycle |
| GLUDC_m | Glutamate decarboxylase | glu_L_m + h_m → 4abut_m + co2_m | 4.1.1.15 | 313744 | Alanine, aspartate and glutamate metabolism |
| ABTArm | 4-aminobutyrate Transaminase, reversible (mitochondrial) | 4abut_m + akg_m ⇌ glu__L_m + sucsal_m | 2.6.1.19 | 69850 | Alanine, aspartate and glutamate metabolism |
| SSALy | Succinate-semialdehyde dehydrogenase (NADP) | h2o_m + nadp_m + sucsal_m → 2.0 h_m + nadph_m + succ_m | 1.2.1.16 | 5880 | Alanine, aspartate and glutamate metabolism |
| ICL | Isocitrate lyase | icit_c → glx_c + succ_c | 4.1.3.1 | 112377 | Glyoxylate cycle |
| MALS | Malate synthase | accoa_c + glx_c + h2o_c → coa_c + h_c + mal_L_c | 2.3.3.9 | 73436 | Glyoxylate cycle |
| MDH | Malate dehydrogenase | mal_L_c + nad_c ⇌ h_c + nadh_c + oaa_c | 1.1.1.37 | 3988, 5696 | Glyoxylate cycle |
| CSc | Citrate synthase | accoa_c + h2o_c + oaa_c → cit_c + coa_c + h_c | 2.3.3.1 | 47067 | Glyoxylate cycle |
| ME1 | Malic enzyme (NAD) | mal_L_c + nad_c → pyr_c + co2_c + nadh_c | 1.1.1.38 | 72728 | Pyruvate metabolism |
| ME2 | Malic enzyme (NADP) | mal_L_c + nadp_c → pyr_c + co2_c + nadph_c | 1.1.1.38 | 72728 | Pyruvate metabolism |
| ACITL | ATP-citrate lyase | atp_c + cit_c + coa_c → accoa_c + adp_c + oaa_c + pi_c | 2.3.3.8 | 5447, 5446 | TCA cycle |
| PYRDC | Pyruvate decarboxylase | h_c + pyr_c → acald_c + co2_c | 4.1.1.1 | 72782, 70370 | Glycolysis |
| ALD | Aldehyde dehydrogenase (acetaldehyde, NAD) | acald_c + h2o_c + nad_c → ac_c + 2.0 h_c + nadh_c | 1.2.1.3 | 767, 314464, 6707, 291711, 5547 | Glycolysis |
| ACS | Acetyl-CoA synthase | ac_c + atp_c + coa_c → accoa_c + amp_c + ppi_c | 6.2.1.1 | 2524 | Gluconeogenesis |
| ACCOAC | Acetyl-CoA carboxylase | accoa_c + atp_c + hco3_c → adp_c + h_c + malcoa_c + pi_c | 6.4.1.2 | 72701 | Fatty acid biosynthesis |
| FAS160 | Fatty-acyl-CoA synthase (n-C16:0CoA) | accoa_c + 21 h_c + 7 malcoa_c + 14 nadph_c → 7 co2_c + 7 coa_c + 7 h2o_c + 14 nadp_c + pmtcoa_c | 2.3.1.86 | 6225 | Fatty acid biosynthesis |
| FAS180 | Fatty-acyl-CoA synthase (n-C18:0CoA) | accoa_c + 24 h_c + 8 malcoa_c + 16 nadph_c → 8 co2_c + 8 coa_c + 8 h2o_c + 16 nadp_c + stcoa_c | 2.3.1.86 | 6225 | Fatty acid biosynthesis |
| DESAT18 | Stearoyl-CoA desaturase (n-C18:0CoA→ n-C18:1CoA) | h_c + nadph_c + o2_c + stcoa_c → 2.0 h2o_c + nadp_c + odecoa_c | 1.14.19.1 | 2700 | Fatty acid metabolism |
| G3PAT160_1 | Glycerol-3-phosphate: acyl-CoA acyltransferase (16:0) | glyc3p_c + pmtcoa_c → 1hdecg3p_c + coa_c | 2.3.1.15 | 68496 | Glycerolipid metabolism |
| AGLPAT160 | 1-acyl-sn-glycerol-3-phosphate acyltransferase (1-16:0, 2-18:1) | 1hdecg3p_c + odecoa_c → pa _c + coa_c | 2.3.1.51 | 237409, 6426 | Glycerolipid metabolism |
| PAPA | Phosphatidate phosphatase (1-16:0, 2-18:1) | h2o_c + pa → coa_c + dag | 3.1.3.4 | 3216, 49055, 80078 | Glycerolipid metabolism |
| DAGT | Diacylglycerol acyltransferase (1-16:0, 2-18:1, 3-18:1) | odecoa_c + dag160181 → coa_c + tag | 2.3.1.20, 2.3.1.22, 2.3.1.26 | 6231, 954, 166982 | Glycerolipid metabolism |
| GLUDy | Glutamate dehydrogenase（NADP） | akg_c + h_c + nadph_c + nh4_c → glu_L_c + h2o_c + nadp_c | 1.4.1.4 | 72563 | Nitrogen metabolism |
| GLUDxi | Glutamate dehydrogenase（NAD） | glu_L_c + h2o_c + nad_c → akg_c + h_c + nadh_c + nh4_c | 1.4.1.2 | 75567 | Nitrogen metabolism |
| GLUSy | Glutamate synthase (NADPH) | akg_c + gln_L_c + h_c + nadph_c → 2.0 glu_L_c + nadp_c | 1.4.1.13 | 3809 | Nitrogen metabolism |
| GLNS | Glutamine synthetase | atp_c + glu_L_c + nh4_c → adp_c + gln_L_c + h_c + pi_c | 6.3.1.2 | 69966 | Nitrogen metabolism |
| SADT | Sulfate adenylyltransferase | atp_c + h_c + so4_c → aps_c + ppi_c | 2.7.7.4 | 1334 | Sulfur metabolism |
| ADSK | Adenylyl-sulfate kinase | aps_c + atp_c → adp_c + h_c + paps_c | 2.7.1.25 | 68607 | Sulfur metabolism |
| PAPSR | Phosphoadenylyl-sulfate reductase(thioredoxin) | paps_c + trdrd_c → 2.0 h_c + pap_c + so3_c + trdox_c | 1.8.4.8 | 68609 | Sulfur metabolism |
| TRDR | Thioredoxin reductase (NADPH) | h_c + nadph_c + trdox_c → nadp_c + trdrd_c | 1.8.1.9 | 404, 70253 | Sulfur metabolism |
| BPNT | 3',5'-bisphosphate nucleotidase | h2o_c + pap_c → amp_c + pi_c | 3.1.3.7 | 588 | Sulfur metabolism |
| SULR | Sulfite reductase (NADPH2) | 5.0 h_c + 3.0 nadph_c + so3_c ⇌ 3.0 h2o_c + h2s_c + 3.0 nadp_c |  |  | Sulfur metabolism |
| BGCELLB | Beta glucosidase | h2o_c + cellb_c ⇌ 2.0 glc__D_c |  |  | Cytosolic reactions |
| XYLR | Xylose reductase | h_c + nadph_c + xyl_D_c → nadp_c + xylt_c | 1.1.1.21 | 4809 | Pentose and glucuronate interconversions |
| XYLTD_D | Xylitol dehydrogenase D xyulose forming | nad_c + xylt_c → h_c + nadh_c + xylu_D_c | 1.1.1.14 | 3740, 72797 | Pentose and glucuronate interconversions |
| XYLK | Xylulokinase | atp_c + xylu_D_c → adp_c + h_c + xu5p_D_c | 2.7.1.17 | 6746 | Pentose and glucuronate interconversions |
| GLYK | Glycerol kinase | atp_c + glyc_c → adp_c + glyc3p_c + h_c | 2.7.1.30 | 332345 | Glycerolipid metabolism |
| G3PDm | Glycerol-3-phosphate dehydrogenase (FAD), mitochondrial | glyc3p_m + fad_m → dhap_m + fadh2_m | 1.1.99.5 | 72144 | Glycerophospholipid metabolism |
| NATPm | NADH dehydrogenase and ATP synthase | 3 h_c + 2.5 adp_m + 2.5 pi_m + nadh_m + 0.5 h_m + 0.5 o2_m → 2.5 atp_m + 3.5 h2o_m + nad_m | 1.6.99.3, 1.10.2.2, 1.9.3.1, 3.6.3.14 | 73435, 60614, 68269, 42388, 112667, 3376, 70538, 47735, 3895, 5045, 54407, 67449, 149291, 90916, 448, 2781, 4281, 4471, 4904, 32736, 40904, 52074, 64406, 71403, 72935, 86782, 4502, 75804 | Oxidative phosphorylation |
| NATPc | NADH dehydrogenase and ATP synthase | h_c + 1.5 adp_m + 1.5 pi_m + nadh_c + 1.5 h_m + 0.5 o2_m → 1.5 atp_m + 2.5 h2o_m + nad_c | 1.6.99.3, 1.10.2.2, 1.9.3.1, 3.6.3.14 | 113845, 4310, 42388, 112667, 3376, 70538, 47735, 3895, 5045, 54407, 67449, 149291, 90916, 448, 2781, 4281, 4471, 4904, 32736, 40904, 52074, 64406, 71403, 72935, 86782, 4502, 75804 | Oxidative phosphorylation |
| FATPm | Succinate dehydrogenase and ATP synthase | 1.5 adp_m + 1.5 pi_m + fadh2_m + 1.5 h_m + 0.5 o2_m → 1.5 atp_m + 2.5 h2o_m + fad_m | 1.3.5.1, 1.10.2.2, 1.9.3.1, 3.6.3.14 | 28592, 58405, 42388, 112667, 3376, 70538, 47735, 3895, 5045, 54407, 67449, 149291, 90916, 448, 2781, 4281, 4471, 4904, 32736, 40904, 52074, 64406, 71403, 72935, 86782, 4502, 75804 | Oxidative phosphorylation |
| BIOR | Biomass pseudoreaction | 25.4 atp_c + 2.4 accoa_c + oaa_c + 1.1 akg_m + 9 nadph_c + 0.6 3pg_c + 0.32 r5p_c + 2.2 nadph_m + 1.6 nad_c + 0.6 nad_m + 0.3 accoa_m + 1.8 pyr_c + 0.32 e4p_c + 0.62 pep_c + 0.153 h2s_c + 0.45 f6p_c + 2.5 g6p_c + 0.1 glyc3p_c + 5.19 glu_L_c + 0.46 gln_L_c + 25.4 h2o_c + 1.6 h_m = 25.4 adp_c + 9 nadp_c + bio_c + 2.2 nadp_m + 1.6 nadh_c + 0.6 nadh_m + 25.4 pi_c + 18 h_c + 0.3 coa_m + 2.4 coa_c |  |  | Growth |
| ATPM | ATP maintenance requirement | atp_c + h2o_c → adp_c + h_c + pi_c |  |  | Cytosolic reactions |
| ATPS | ATPase | atp_c + h2o_c → adp_c + pi_c + h_e | 3.6.3.6 | 3900, 5220, 117465 | Cytosolic reactions |
| ADK1 | Adenylate kinase | amp_c + atp_c ⇌ 2.0 adp_c | 2.7.4.3 | 2516, 4094 | Cytosolic reactions |
| PPA | Inorganic diphosphatase | h2o_c + ppi_c → h_c + 2.0 pi_c | 3.6.1.1 | 4257 | Cytosolic reactions |
| HCO3E | HCO3 equilibration reaction | co2_c + h2o_c ⇌ h_c + hco3_c |  |  | Spontaneous reaction |
| CITMALtm | Citrate transport | cit_m + mal_L_c ⇌ cit_c + mal_L_m |  | 73737, 81859 | Transport |
| CITtm | Citrate transport mitochondrial | cit_c + icit_m ⇌ icit_c + cit_m |  | 73737, 81859 | Transport |
| SUCFUMtm | Succinate fumarate transport mitochondrial | succ_c + fum_m → fum_c + succ_m |  | 3267 | Transport |
| CRNAOT | Carnitine O-acetyltransferase and carnithine-acetylcarnithine carrier | accoa_c + coa_m → accoa_m + coa_c | 2.3.1.7 | 423, 105066, 67775, 72342 | Transport |
| AKGMALtm | 2-oxoglutarate/L-malate exchange | akg_m + mal_L_c ⇌ akg_c + mal_L_m |  | 67687 | Transport |
| PYRtm | Pyruvate transport | h_c + pyr_c ⇌ h_m + pyr_m |  | 73446, 103731 | Transport |
| OAAtm | Oxaloacetate transport | h_c + oaa_c ⇌ h_m + oaa_m |  | 976 | Transport |
| SUCCtm | Succinate transport | pi_m + succ_c ⇌ pi_c + succ_m |  | 72902 | Transport |
| MALtm | Malate transport | mal_L_c + pi_m ⇌ mal_L_m + pi_c |  | 72902 | Transport |
| ATPtm | ADP/ATP transporter | adp_c + atp_m ⇌ adp_m + atp_c |  | 3617 | Transport |
| CELLBt | Cellobiose transport | cellb_e ⇌ cellb_c |  | 120451 | Transport |
| NNshuttle | NAD-NADH shuttle | nad_m + nad_c → nadh_c + nadh_m |  |  | Transport |
| H2Otm | H2O transport diffusion | h2o_c ⇌ h2o_m |  |  | Transport |
| CO2tm | CO2 transport diffusion | co2_c ⇌ co2_m |  |  | Transport |
| O2tm | O2 transport diffusion | o2_c ⇌ o2_m |  |  | Transport |
| GLCt | D-glucose transport | glc_D_e ⇌ glc_D_c |  |  | Transport |
| ACt | Acetate transport | ac_e ⇌ ac_c |  |  | Transport |
| XYLt | D-Xylose transport | xyl_D_e ⇌ xyl_D_c |  |  | Transport |
| GLYCt | Glycerol transport via channel | glyc_c → glyc_e |  |  | Transport |
| GLYCt2 | Glycerol transport via symport | glyc_e + h_e → glyc_c + h_c |  |  | Transport |
| H2Ot | H2O transport diffusion | h2o_e ⇌ h2o_c |  |  | Transport |
| PIt | Phosphate reversible transport via symport | h_e + pi_e ⇌ pi_c + h_c |  |  | Transport |
| PItm | Phosphate transport | h_c + pi_c ⇌ h_m + pi_m |  |  | Transport |
| CO2t | CO2 transport | co2_e ⇌ co2_c |  |  | Transport |
| NH4t | Ammonium transport | nh4_e ⇌ nh4_c |  |  | Transport |
| O2t | O2 transport | o2_e ⇌ o2_c |  |  | Transport |
| SO4t | Sulfate transport | so4_e ⇌ so4_c |  |  | Transport |
| Ht | H+ diffusion | h_e ⇌ h_c |  |  | Transport |
| Htm | H+ transport | h_c ⇌ h_m |  |  | Transport |
| GLYC3Ptm | Glycerol-3-phosphate shuttle | glyc3p_c ⇌ glyc3p_m |  |  | Transport |
| DHAPtm | Dihydroxyacetone phosphate transport mitochondrial | dhap_m ⇌ dhap_c |  |  | Transport |
| EX_glc_D_e | D-glucose exchange | glc_D_e ⇌ |  |  | exchange |
| EX_cellb_e | Cellobiose exchange | cellb_c ⇌ |  |  | exchange |
| EX_ac_e | Acetate exchange | ac_e ⇌ |  |  | exchange |
| EX_xyl_D_e | D-Xylose exchange | xyl_D_e ⇌ |  |  | exchange |
| EX_glyc_e | Glycerol exchange | glyc_e ⇌ |  |  | exchange |
| EX_h_e | H+ exchange | h_e ⇌ |  |  | exchange |
| EX_h2o_e | H2O transport exchange | h2o_e ⇌ |  |  | exchange |
| EX_pi_e | Phosphate exchange | pi_e ⇌ |  |  | exchange |
| EX_co2_e | CO2 exchange | co2_e ⇌ |  |  | exchange |
| EX_nh4_e | Ammonium exchange | nh4_e ⇌ |  |  | exchange |
| EX_o2_e | O2 exchange | o2_e ⇌ |  |  | exchange |
| EX_so4_e | Sulfate exchange | so4_e ⇌ |  |  | exchange |
| Bio_c_Ex | Growth | bio_c → |  |  | Efflux |
| Tag_c_Ex | Triglyceride export | tag_c → |  |  | Efflux |
| GAPDy | Hypothetical NADP-glyceraldehyde-3-phosphate dehydrogenase | g3p_c + nadp_c + pi_c ⇌ 13dpg_c + h_c + nadph_c |  |  | Cytosolic reactions |
| ALDy | Hypothetical NADP-aldehyde dehydrogenase | acald_c + h2o_c + nadp_c → ac_c + 2.0 h_c + nadph_c |  |  | Cytosolic reactions |
| NADTRHD | Hypothetical NAD-transhydrogenase | nad_c + nadph_c ⇌ nadh_c + nadp_c |  |  | Cytosolic reactions |
| ICDHxmr | Hypothetical reversible isocitrate dehydrogenase | icit_m + nad_m ⇌ akg_m + co2_m + nadh_m |  |  | Cytosolic reactions |
| FAS160* | Hypothetical fatty acid synthesis reaction by reversal of β-oxidation | 7 h_c + 7 nadh_c + 7 fadh2_m + 8 accoa_c → 7 nad_c + 7 fad_m + 1 pmtcoa_c + 7 coa_c |  |  | Cytosolic reactions |
| FAS180* |  | 8 h_c + 8 nadh_c + 8 fadh2_m + 8 accoa_c → 8 nad_c + 8 fad_m + 1 stcoa_c + 7 coa_c |  |  | Cytosolic reactions |

Table S2 Metabolites in the small-scale metabolic model of *L. starkeyi* NRRL Y-11557

| Abbreviation | Name | Formula | Abbreviation | Name | Formula |
| --- | --- | --- | --- | --- | --- |
| atp_c | ATP | C10H12N5O13P3 | accoa_c | Acetyl-CoA | C23H34N7O17P3S |
| atp_m | ATP | C10H12N5O13P3 | fadh2_m | Flavin adenine dinucleotide reduced | C27H33N9O15P2 |
| adp_c | ADP | C10H12N5O10P2 | 3pg_c | 3-Phospho-D-glycerate | C3H4O7P |
| adp_m | ADP | C10H12N5O10P2 | icit_c | Isocitrate | C6H5O7 |
| g6p_c | D-Glucose 6-phosphate | C6H11O9P | icit_m | Isocitrate | C6H5O7 |
| glc_D_c | D-Glucose | C6H12O6 | akg_c | 2-Oxoglutarate | C5H4O5 |
| glc_D_e | D-Glucose | C6H12O6 | akg_m | 2-Oxoglutarate | C5H4O5 |
| cellb_e | Cellobiose | C12H22O11 | succoa_m | Succinyl-CoA | C25H35N7O19P3S |
| cellb_c | Cellobiose | C12H22O11 | succ_c | Succinate | C4H4O4 |
| h_c | H+ | H | succ_m | Succinate | C4H4O4 |
| h_m | H+ | H | sucsal_m | Succinic semialdehyde | C4H5O3 |
| h_e | H+ | H | fum_c | Fumarate | C4H2O4 |
| f6p_c | D-Fructose 6-phosphate | C6H11O9P | fum_m | Fumarate | C4H2O4 |
| fdp_c | D-Fructose 1,6-bisphosphate | C6H10O12P2 | mal_L_c | L-Malate | C4H4O5 |
| h2o_c | H2O | H2O | mal_L_m | L-Malate | C4H4O5 |
| h2o_m | H2O | H2O | hco3_c | Bicarbonate | HCO3 |
| h2o_e | H2O | H2O | hco3_m | Bicarbonate | HCO3 |
| pi_c | Phosphate | HO4P | glx_c | Glyoxylate | C2HO3 |
| pi_m | Phosphate | HO4P | glu_L_c | L-Glutamate | C5H8NO4 |
| pi_e | Phosphate | HO4P | glu_L_m | L-Glutamate | C5H8NO4 |
| dhap_c | Dihydroxyacetone phosphate | C3H5O6P | gln_L_c | L-Glutamine | C5H10N2O3 |
| dhap_m | Dihydroxyacetone phosphate | C3H5O6P | 4abut_m | 4-Aminobutanoate | C4H9NO2 |
| g3p_c | Glyceraldehyde 3-phosphate | C3H5O6P | acald_c | Acetaldehyde | C2H4O |
| 13dpg_c | 3-Phospho-D-glyceroyl phosphate | C3H4O10P2 | ac_c | Acetate | C2H3O2 |
| nad_c | Nicotinamide adenine dinucleotide | C21H26N7O14P2 | ac_e | Acetate | C2H3O2 |
| nad_m | Nicotinamide adenine dinucleotide | C21H26N7O14P2 | amp_c | AMP | C10H12N5O7P |
| nadh_c | Nicotinamide adenine dinucleotide - reduced | C21H27N7O14P2 | ppi_c | Diphosphate | HO7P2 |
| nadh_m | Nicotinamide adenine dinucleotide - reduced | C21H27N7O14P2 | malcoa_c | Malonyl CoA | C24H34N7O19P3S |
| glyc3p_c | Glycerol 3-phosphate | C3H7O6P | pmtcoa_c | Palmitoyl-CoA | C37H62N7O17P3S |
| glyc3p_m | Glycerol 3-phosphate | C3H7O6P | stcoa_c | Stearoyl-CoA (n-C18:0CoA) | C39H66N7O17P3S |
| fad_m | Flavin adenine dinucleotide oxidized | C27H31N9O15P2 | odecoa_c | Octadecenoyl-CoA (n-C18:1CoA) | C39H64N7O17P3S |
| 1hdecg3p_c | 1-hexadecanoyl-sn-glycerol 3-phosphate | C19H37O7P | coa_c | Coenzyme A | C21H32N7O16P3S |
| pa_c | Phosphatidate(1-16:0,2-18:1) | C37H69O8P | coa_m | Coenzyme A | C21H32N7O16P3S |
| dag_c | Diglyceride (1-16:0,2-18:1) | C37H70O5 | oaa_c | Oxaloacetate | C4H2O5 |
| tag_c | Triglyceride (1-16:0,2-18:1,3-18:1) | C55H102O6 | oaa_m | Oxaloacetate | C4H2O5 |
| o2_c | O2 | O2 | cit_c | Citrate | C6H5O7 |
| o2_m | O2 | O2 | cit_m | Citrate | C6H5O7 |
| o2_e | O2 | O2 | aps_c | Adenosine 5'-phosphosulfate | C10H12N5O10PS |
| 2pg_c | D-Glycerate 2-phosphate | C3H4O7P | accoa_m | Acetyl-CoA | C23H34N7O17P3S |
| pep_c | Phosphoenolpyruvate | C3H2O6P | so4_c | Sulfate | SO4 |
| pyr_c | Pyruvate | C3H3O3 | so4_e | Sulfate | SO4 |
| pyr_m | Pyruvate | C3H3O3 | h2s_c | Hydrogen sulfide | H2S |
| 6pgl_c | 6-phospho-D-glucono-1,5-lactone | C6H9O9P | so3_c | Sulfite | SO3 |
| nadp_c | Nicotinamide adenine dinucleotide phosphate | C21H25N7O17P3 | paps_c | 3'-Phosphoadenylyl sulfate | C10H11N5O13P2S |
| nadp_m | Nicotinamide adenine dinucleotide phosphate | C21H25N7O17P3 | pap_c | Adenosine 3',5'-bisphosphate | C10H11N5O10P2 |
| nadph_c | Nicotinamide adenine dinucleotide phosphate - reduced | C21H26N7O17P3 | trdox_c | Oxidized thioredoxin | X |
| nadph_m | Nicotinamide adenine dinucleotide phosphate - reduced | C21H26N7O17P3 | trdrd_c | Reduced thioredoxin | XH2 |
| 6pgc_c | 6-Phospho-D-gluconate | C6H10O10P | nh4_c | Ammonium | NH4 |
| co2_c | CO2 | CO2 | nh4_e | Ammonium | NH4 |
| co2_m | CO2 | CO2 | xyl_D_c | D-Xylose | C5H10O5 |
| co2_e | CO2 | CO2 | xyl_D_e | D-Xylose | C5H10O5 |
| ru5p_D_c | D-Ribulose 5-phosphate | C5H9O8P | xylt_c | Xylitol | C5H12O5 |
| r5p_c | Alpha-D-Ribose 5-phosphate | C5H9O8P | xylu_D_c | D-Xylulose | C5H10O5 |
| xu5p_D_c | D-Xylulose 5-phosphate | C5H9O8P | glyc_c | Glycerol | C3H8O3 |
| s7p_c | Sedoheptulose 7-phosphate | C7H13O10P | glyc_e | Glycerol | C3H8O3 |
| e4p_c | D-Erythrose 4-phosphate | C4H7O7P | bio_c | Biomass |  |
